# Ginsenoside Rg1 Mitigates Porcine Intestinal Tight Junction Disruptions Induced by LPS through the p38 MAPK/*NLRP3* Inflammasome Pathway

**DOI:** 10.3390/toxics10060285

**Published:** 2022-05-27

**Authors:** Jian Kang, Yanhong Zhou, Chunyang Zhu, Tian Ren, Yong Zhang, Longfei Xiao, Binghu Fang

**Affiliations:** 1 College of Veterinary Medicine, South China Agricultural University, Guangzhou 510630, China; kj89260541@163.com (J.K.); 18312670440@163.com (Y.Z.); cy0518vet@163.com (C.Z.); tianhn@163.com (T.R.); 2 College of Veterinary Medicine, Gansu Agricultural University, Lanzhou 730070, China; 13730333886@163.com; 3 Animal Science and Technology College, Beijing University of Agriculture, Beijing 100096, China

**Keywords:** ginsenoside Rg1, LPS, P38, *NLRP3*, IPEC-J2, mice, tight junction

## Abstract

Inflammation leads to porcine tight junction disruption of small intestinal epithelial cells, resulting in intestinal dysfunction. Herein, we established lipopolysaccharide (LPS)-induced in-vivo and in-vitro inflammatory models. The results revealed that LPS induced tight junction disruption in IPEC-J2 cells by downregulating tight-junction-related protein zonula occludens-1 (ZO-1), occludin and claudin-1 expression, while ginsenoside Rg1 rescued such inhibition and abrogated the upregulated expression of phosphorylation p38 MAPK. The p38 MAPK inhibitor (SB203580) showed a similar effect with Rg1 and attenuated the LPS-induced inhibition of ZO-1, occludin and claudin-1 expression, which is consistent with the reduced expression of *NLRP3* inflammasome and *IL-1β*. Furthermore, the specific inhibitors of *NLRP3* and *IL-1β* result in increased expression of tight-junction-related protein, demonstrating that p38 MAPK signaling was associated with Rg1 suppression of tight junction disruption. Besides, LPS treatment decreased the expression of ZO-1, occludin and claudin-1 through p38 MAPK signaling, and caused abnormal morphological changes in murine ileum. Meanwhile, Rg1 attenuated the decreased expression of ZO-1, occludin and claudin-1 and partially alleviated LPS-induced morphological changes in murine ileum. In summary, these findings characterized a novel mechanism by which Rg1 alleviates LPS-induced intestinal tight junction disruption by inhibiting the p38 MAPK-mediated *NLRP3* inflammasome pathway.

## 1. Introduction

Intestinal dysfunction is a critical problem in pork production and can be caused by several factors, including weaning, bacterial or viral infections, and tight junction disruption [1]. One of the most important components of the intestinal barrier is the intestinal tight junction, which functions together with the mucous gel layer and mucosal immune system to provide a physical barrier to the diffusion of pathogens and toxins into the circulatory system from the luminal environment. [2,3]. A number of reports have documented that intestinal dysfunction due to the disruption of tight junctions can cause various intestinal illnesses, including inflammatory bowel disease (IBD), irritable bowel syndrome (IBS), celiac disease [4] and sepsis [5] in pigs, causing huge economic losses to the global porcine industry. As a consequence, the restoration of tight junctions in the intestine is critical to the recovery of intestinal function.

Lipopolysaccharide (LPS) is a class of endotoxins produced by Gram-negative bacteria. LPS is toxic to humans and stimulates several cytokines associated with inflammation. It is believed to be involved in the pathogenesis of numerous disorders, such as endotoxemia/sepsis, multiorgan injuries and liver damage, etc. [6,7]. Previously, it has been demonstrated that LPS was found to induce the intestinal inflammation and destruction of tight junctions [8]. It is, therefore, of great significance to investigate the molecular mechanisms involved in LPS-induced intestinal toxicity. It is well known that p38 MAPK, an important intracellular signal transduction system, is associated with trauma [9], infection [10] and inflammation [11]. Several physiological and pathological responses in mammalian intestines are mediated by the p38 MAPK signaling pathway, such as ischemia-reperfusion (I/R) injury caused by inflammation [12], intestinal disturbances, and oxidative stress [13]. However, the involvement of the p38 MAPK signaling pathway in the disruption of inflammation-mediated tight junctions is still unclear. The NLR family pyrin domain containing 3 (*NLRP3*) inflammasomes are among the protein complexes in the cytosol, which play an important role in microbial infections and in regulating the immune, metabolic and mucosal systems. [14]. The *NLRP3* inflammasome complex consists of the sensor *NLRP3*, the adaptor-apoptosis-associated speck-like protein and the effector protein-caspase-1 [15]. Activated caspase-1 cleaves substrates, such as pro-*IL-1β*, to produce *IL-1β* with biological activity that stimulates inflammation in intestinal epithelial cells [16]. Researchers reported that inflammatory factors can further damage tight junctions in the digestive tract, thus, contributing to the development or deterioration of intestinal diseases [17]. Therefore, it is crucial to investigate active pharmaceutical agents that may prevent intestinal dysfunction.

Ginseng is widely used in traditional Chinese medicine due to its antitumor and anti-inflammatory properties [18,19], which are attributed, primarily, to ginsenosides [20]. Among them, ginsenoside Rg1 (hereinafter referred to as Rg1) is the most active and abundant steroidal saponin, with a similar structure to those of other steroidal hormones [21]. Rg1 has many pharmacological effects, such as antitumor, immuno-modulatory and anti-inflammatory activity [22,23]. A recent study indicated that Rg1 reverses *IL-1β*-mediated downregulation in macrophages associated with the disruption of tight junction proteins [24]. Based on the above-mentioned concerns, this study explored the protective effect of Rg1 on the LPS-induced disruption of tight junctions in IPEC-J2 cells and its underlying mechanisms. Meanwhile, we have also demonstrated that Rg1 regulates intestinal tight junctions and decreases LPS-induced intestinal tight junctions in mice, thus, providing new insights into Rg1’s role in the recovery of intestinal dysfunctions.

## 2. Materials and Methods

LPS, Rg1, MCC950, IL-1Ra and SB 203580 were purchased from MedChemExpress (Shanghai, China). Rg1 and LPS were purchased from Shanghai Macklin Biochemical Co., Ltd. (Shanghai, China).

Specific pathogen-free (SPF) 6-week-old female KM mice (Laboratory Animal Center of South China Agricultural University, China) were kept in single cages under a 12 h light/dark cycle, at temperature (20–25°C) and humidity (40–70%). The animals were provided ad libitum access to food and water for 7 days. Before the experiment, the animals were fasted for 12 h. All the experimental protocols were approved by the Animal Protection and Use Committee of South China Agricultural University.

Thirty-two mice were randomly divided into four groups (eight mice per group). Group 1 was the vehicle control group in which the mice were administered with normal saline intraperitoneally (i.p.). Group 2 was the LPS group in which the mice were i.p. injected with LPS at a dose of 10 mg/kg without Rg1 treatment. Groups 3 and 4 were the treatment groups in which the mice were i.p injected with LPS for three consecutive days at a dose of 10 mg/kg per day, and after daily injection, Rg1 was orally administered at a dose of 10 and 20 mg/kg, respectively. At 24 h after the last administration, all the mice were sacrificed after being anesthetized by i.p. injection with pentobarbital (50 mg/kg), and their ileum samples were collected for further analyses.

PEC-J2 cells (Tongpai Biotechnology Co., LTD, Shanghai, China) were cultured in DMEM/F--12 medium (Gibco, Shanghai, China) with 10% FBS (Gibco, Shanghai, China) and 1% penicillin-streptomycin (Invitrogen, Shanghai, China) at 37°C and 5% CO2. When being cultured to four to eight generations, the cells were seeded in six-well plates (5 × 105 cells/well) and cultured for 24 h, and then treated with LPS (at a final concentration of 0.5, 1 and 2 μg/mL) and/or Rg1(at a final concentration of 10 and 60 μM) at the indicated concentrations for 48 h.

Total RNA was extracted from the tissues and cells with TRIzol reagent (Solarbio, Beijing, China) and immediately reverse transcribed with a Prime Script RT Reagent Kit with gDNA Eraser (TaKaRa, Dalian, China). qPCR was performed on the amplified cDNA with SYBR Green II PCR mix (TaKaRa) and a LightCycler 480 Realtime Detection System (Roche, Basel, Switzerland). The qPCR reactants consisted of 10 L 2 SYBR Green II PCR mix (TaKaRa), 25 μmol/L forward and reverse primers, 2 μL template, and ddH2O to a total volume of 20 μL. The thermocycler was set to 95 °C for 600 s, followed by 40 cycles of 95 °C for 10 s and 60 °C for 30 s. The melting curve was obtained from 65 to 95 °C, increasing by an increment of 0.5 °C every 5 s. The data were analyzed with the 2^−ΔΔCt^ calculation method and *β-actin* as the internal reference. Table 1 lists the sequences of the forward and reverse primers used in this study.

The tissues and cells were collected and washed with ice-cold PBS, and then treated with ice-cold radioimmunoprecipitation assay (RIPA) lysis buffer containing 1 mm phenylmethylsulfonyl fluoride (PMSF). Western blotting analysis was performed as described previously. The primary antibodies were ZO-1 (1:3000 dilution; bs-1329R; Bioss), occludin (1:3000 dilution; bs-10011R; Bioss), claudin-1 (1:1000 dilution; bs-10008R; Bioss), phosphorylated p38 MAPK (α) (hereinafter referred to as p-p38) (1:1000 dilution; bs-0636R; Bioss), p38 MAPK (α) (1:1000 dilution; bsm-33423R; Bioss), *NLRP3* (1:1000 dilution; bs-10021R; Bioss), *IL-1β* (1:1000 dilution; bs-0821R; Bioss) and *β-actin* (1:3000 dilution; bs-0061R; Bioss). HRP-conjugated goat anti-rabbit antibody (1:3000 dilution, bs-0295P-HRP; Bioss) was used as the secondary antibody. The bands were detected with ECL solution and signals were quantified with Image J software.

Ileum was fixed in 10% neutral buffered formalin and embedded in paraffin. The paraffin was sectioned (5 μm thick) for hematoxylin and eosin (H&E) staining to observe the morphological structure. The sections were observed and photographed with the Olympus-DP73 optical microscope (Tokyo, Japan).

Data are expressed as the mean ± standard error of means (SEM). All statistical analyses were performed using the SPSS 16.0 software (Chicago, IL, USA). Data were analyzed by one-way ANOVAs. A *p* value of less than 0.05 is considered statistically significant.

## 3. Results

### 3.1. Effects of Different Concentrations of LPS on ZO-1, Occludin and Claudin-1 Expression in IPEC-J2 Cells

**Figure 1:**
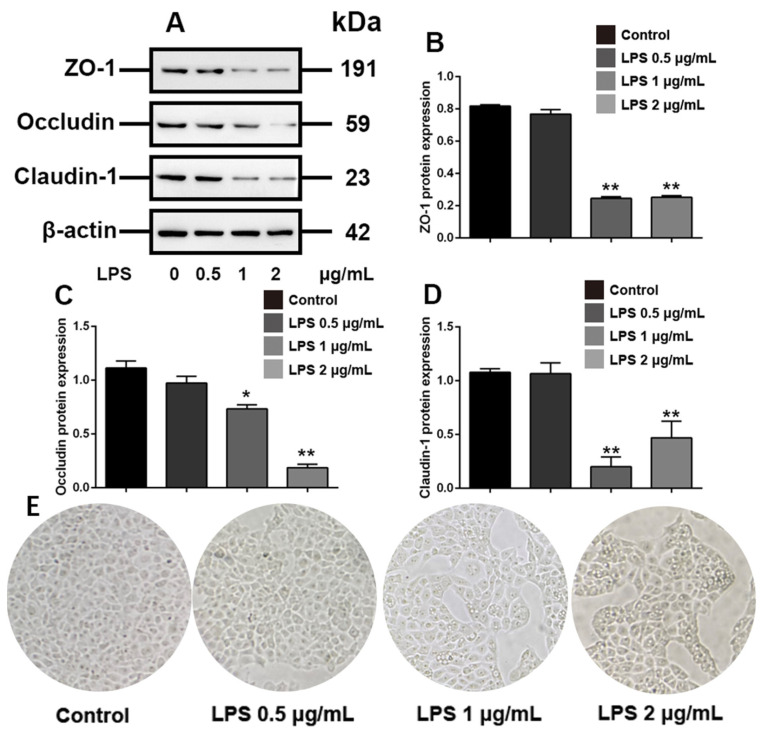
LPS disrupts tight junctions in IPEC-J2 cells. IPEC-J2 cells were treated with vehicle or LPS at the indicated concentrations for 48 h. (**A**) Western blot analyses of tight-junction-related proteins; effect of LPS on ZO-1 (**B**), occludin (**C**) and claudin-1 (**D**) in IPEC-J2 cells; (**E**) morphology of IPEC-J2 (400 × total magnification). β-Actin was used as an internal control. Blots are representative of three independent experiments with densitometry showing the mean ± s.d. * *p* < 0.05 versus control and ** *p* < 0.01 versus control.

LPS is commonly used as an inflammatory-mimetic agent. We aimed to assess tight junction disruption induced by LPS-mediated inflammation in IPEC-J2 cells. We initially set three doses to determine the LPS concentration required to cause significant damage to the tight junctions of IPEC-J2 cells. The results showed that LPS at doses below 1 µg/mL for 48 h did not significantly reduce the expression of the tight junction proteins ZO-1, occludin, and claudin-1 (*p* > 0.05) and 2 μg/mL LPS showed more obvious inhibitory effects on the expression of occludin (*p* < 0.01) (Figure 1A–D). In addition, discrete cell arrangement and more cell gaps were observed in IPEC-J2 treated with 2 μg/mL LPS (Figure 1E). These results indicated that LPS with a concentration of 2 μg/mL can induce obvious tight junction disruption of IPEC-J2 cells.

### 3.2. Rg1 Inhibited LPS-Induced Morphological Damage of Ipec-j2 Cells via Downregulating ZO-1, Occludin and Claudin-1 Expression

**Figure 2:**
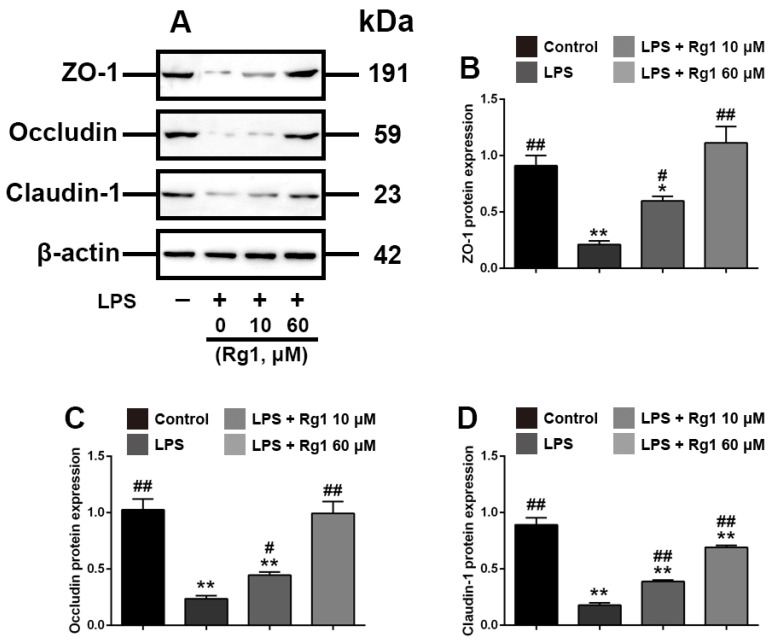
Rg1 inhibits LPS-induced tight junction disruption in IPEC-J2 cells in vitro. IPEC-J2 cells were treated with vehicle, Rg1 or LPS at the indicated concentrations for 48 h. (**A**) Western blot analyses of tight-junction-related proteins; effect of Rg1 on ZO-1 (**B**), occludin (**C**) and claudin-1 (**D**) in IPEC-J2 cells. β-Actin was used as an invariant control for equal loading. Blots are representative of three independent experiments with densitometry, showing the mean ± s.d. * *p* < 0.05 versus control and ** *p* < 0.01 versus control. # *p* < 0.05 versus LPS and ## *p* < 0.01 versus LPS.

We subsequently investigated the effects of different doses of Rg1 on LPS-induced tight junction disruptions in IPEC-J2 cells. As shown in Figure 2, Rg1 at a dose of 10 and 60 μM significantly attenuated the inhibition of ZO-1, occludin and claudin-1 proteins’ expression (*p* < 0.05) and partially ameliorated LPS-induced morphological damage in IPEC-J2 cells. Taken together, these results demonstrated that Rg1 alleviates LPS-induced intestinal tight junction destruction in IPEC-J2 cells.

### 3.3. Rg1 Inhibits LPS-Induced Tight Junction Disruption in IPEC-J2 Cells by Interfering with P38 MAPK Signaling Pathway

**Figure 3:**
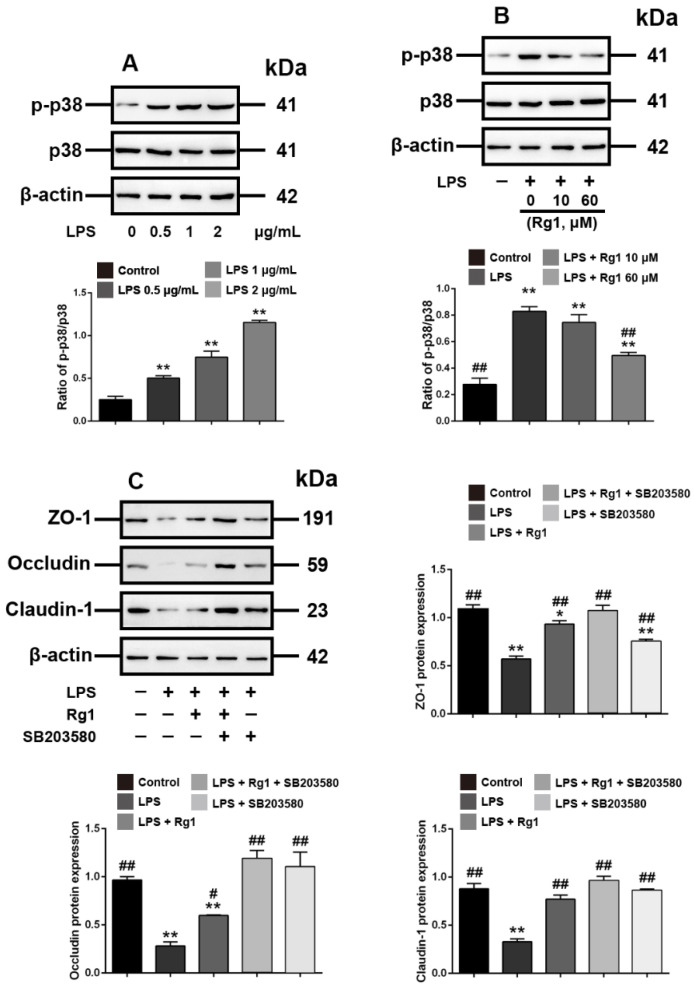
Interference with p38 MAPK signaling is associated with Rg1 inhibition of LPS-induced tight junction disruption in IPEC-J2 cells in vitro. IPEC-J2 cells were treated with vehicle and LPS at the indicated concentrations for 48 h. (**A**) Western blot analyses of p38 MAPK signaling molecules. IPEC-J2 cells were treated with vehicle, LPS (2 μg/mL) and Rg1 at the indicated concentrations for 48 h; (**B**) Western blot analyses of p38 MAPK signaling molecules. IPEC-J2 cells were treated with vehicle, LPS (2 μg/mL), SB203580 (10 μM), and Rg1 (60 μM) for 48 h; (**C**) Western blot analyses of tight-junction-related proteins. β-Actin was used as an invariant control for equal loading. Blots are representative of three independent experiments with densitometry showing the mean ± s.d. * *p* < 0.05 versus control and ** *p* < 0.01 versus control. # *p* < 0.05 versus LPS and ## *p* < 0.01 versus LPS.

We next explored the molecular events underlying the Rg1 inhibition of LPS-induced tight junction disruption in IPEC-J2 cells. p38 MAPK signaling has been demonstrated to have a key role in the tight junction of various tissues [25,26]. We herein found that the phosphorylation of p38 MAPK was upregulated in LPS-treated IPEC-J2 cells (Figure 3A). Rg1 reduced the phosphorylation of p38 MAPK dose dependently, indicating Rg1 interference with the p38 MAPK signaling in LPS-stimulated IPEC-J2 cells (Figure 3B). To evaluate whether p38 MAPK signaling activation is involved in Rg1-inhibited tight junction destruction in LPS-stimulated IPEC-J2 cells, SB203580, a p38 MAPK inhibitor, was used. As shown in Figure 3C, Rg1 effects under LPS treatment were similar to those observed when IPEC-J2 cells were exposed to SB203580, and the combined treatment of Rg1 and SB203580 decreased the expression of ZO-1, occludin and claudin-1. Hence, Rg1 inhibition of LPS-induced tight junction disruption in IPEC-J2 cells was associated with inhibition of the p38 MAPK signaling pathway in vitro.

### 3.4. Rg1 Inhibits the mRNA and Protein Expression of NLRP3 and IL-1β through p38 MAPK Signaling Pathway

**Figure 4:**
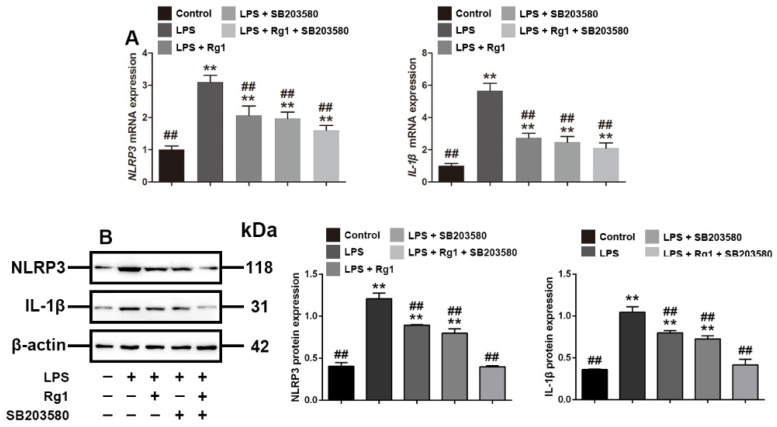
Rg1 regulates *NLRP3* and *IL-1β* through p38 MAPK signaling in IPEC-J2 cells treated with LPS in vitro. PEC-J2 cells were treated with vehicle, LPS (2 μg/mL), SB203580 (10 μM), and Rg1 (60 μM) for 48 h. (**A**) mRNA level analyses of *NLRP3* and *IL-1β*; (**B**) Western blot analyses of *NLRP3* and *IL-1β*. β-Actin was used as an invariant control for equal loading. Blots are representative of three independent experiments with densitometry showing the mean ± s.d. ** *p* < 0.01 versus control. ## *p* < 0.01 versus LPS.

To verify that Rg1 reversed LPS-induced tight junction disruption through p38 MAPK signaling, we evaluated the effects of Rg1 and SB203580 on *NLRP3* and *IL-1β*, which belong to the downstream proteins of p38 MAPK signaling, as demonstrated previously [27,28]. The Q-PCR and Western blot results showed (Figure 4) that LPS significantly enhanced the mRNA and protein expression of *NLRP3* and *IL-1β*, while Rg1 treatment significantly reversed the increased expression (*p* < 0.01). We further used SB203580 to verify that the expression of *NLRP3* and *IL-1β* is associated with p38 MAPK signaling, and the result showed that Rg1 had a similar effect with SB203580 in reducing the expression of *NLRP3* and *IL-1β* by inhibiting p38 MAPK signaling.

### 3.5. Effects of MCC950 and IL-1Ra on Expression of ZO-1, Occludin, and Claudin-1 in LPS-Treated IPEC-J2 Cells

**Figure 5:**
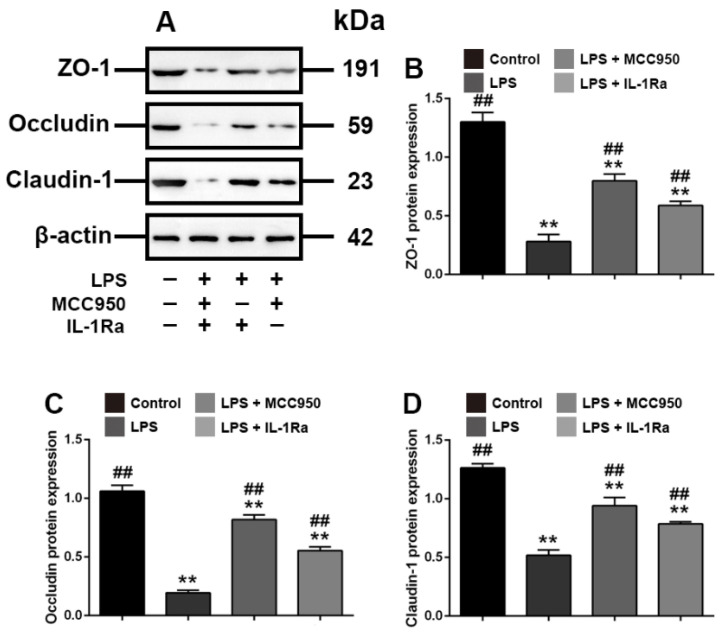
Interference with *NLRP3* and *IL-1β* is associated with tight junction in IPEC-J2 cells in vitro. PEC-J2 cells were treated with vehicle, LPS (2 μg/mL), MCC950 (10 μm) and IL-1Ra (10 ng/mL) for 48 h. Western blot analyses of tight-junction-related proteins. (**A**) Western blot analyses of tight-junction-related proteins; effect of LPS on ZO-1 (**B**), occludin (**C**) and claudin-1 (**D**) in IPEC-2 cells. β-Actin was used as an invariant control for equal loading. Blots are representative of three independent experiments with densitometry showing the mean ± s.d. ** *p* < 0.01 versus control. ## *p* < 0.01 versus LPS.

Subsequently, to examine the effects of the increased expression of *NLRP3* and *IL-1β* on tight junctions, we investigated the effects of the specific inhibitors of *NLRP3* and *IL-1β*, MCC950 and IL-1Ra, on the expression of ZO-1, occludin and claudin-1. The results showed that the MCC950 and IL-1Ra-mediated inhibition of both *NLRP3* and *IL-1β* reversed the decreased expression of ZO-1, occludin and claudin-1 caused by LPS (*p* < 0.01) (Figure 5). Thus, these in-vitro data indicated that Rg1 inhibited tight junction disruption associated with LPS-caused intestinal dysfunction in IPEC-J2 cells.

### 3.6. Rg1 Inhibits Tight Junction Disruption via P38 MAPK Signaling in Mice

**Figure 6:**
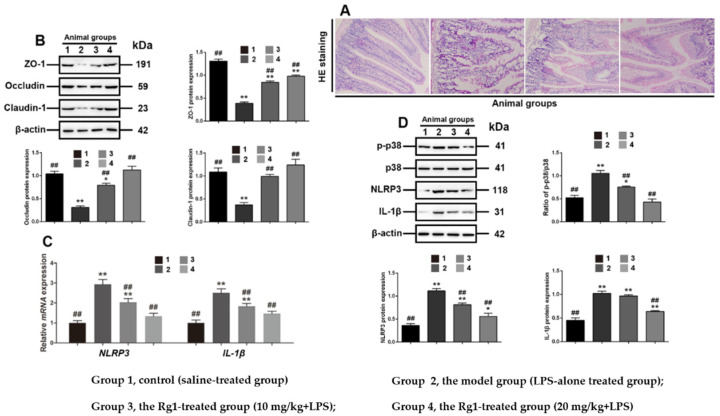
Rg1 inhibits tight junction disruption associated with interference with p38 MAPK signaling in murine intestinal dysfunction caused by LPS. Experimental groups including Group 1, control (saline-treated group); Group 2, the model group (LPS-alone-treated group); Group 3, the Rg1-treated group (10 mg/kg + LPS); and Group 4, the Rg1-treated group (20 mg/kg + LPS). Ileum sections were stained with HE for histological assessment. (**A**) Representative views are shown (original magnification × 20); (**B**) Western blot analyses of tight-junction-related proteins. Western blot analyses of tight-junction-related proteins; (**C**) mRNA level analyses of *NLRP3* and *IL-1β*; (**D**) Western blot analyses of p38 MAPK signaling-related proteins. β-Actin was used as an invariant control for equal loading. Blots are representative of three independent experiments with densitometry showing the mean ± s.d. * *p* < 0.05 versus control and ** *p* < 0.01 versus control. ## *p* < 0.01 versus LPS.

The effects of Rg1 on tight junction disruption in intestinal dysfunction were examined in LPS-treated mice. Histological examination showed that Rg1 partially ameliorated the abnormal pathological changes in the small intestine (Figure 6A). In addition, Western blot analyses further showed that Rg1 dose dependently and partially reduced *NLRP3*, *IL-1β* expression and the phosphorylation of p38 MAPK in the experimental animals (Figure 6C), and restored the level of ZO-1, occludin and claudin-1 protein (Figure 6B), consistent with the results obtained in LPS-treated IPEC-J2 cells. Thus, it has been further confirmed that Rg1 protected the intestinal tight junctions associated with p38 MAPK-dependent *NLRP3* signaling pathway in LPS-induced intestinal dysfunction.

## 4. Discussion

Ginsenosides are considered as key constituents for ginseng function against several diseases, among which Rg1 has attracted much attention because of its antitumor, immunomodulatory and anti-inflammatory effects [29,30,31]. In this study, we found that Rg1 can restore the tight junction dysfunction of porcine intestines by inhibiting *NLRP3*/*IL-1β* regulated by p38 MAPK signaling to some extent.

Tight junctions shape the continuous intercellular barrier, which acts as the first physical barrier against a variety of pathogens and maintains homeostasis in the gastrointestinal tract [32]. The functions of mammalian intestinal tight junctions are determined by several types of proteins, namely junctional adhesion molecules (JAMs), occludin and claudins, and other cytoplasmic tight junction proteins, including ZO-1 and claudins [33]. A recent study pointed out that intestinal barrier dysfunction was reflected by the downregulation of the expression of ZO-1, occludin and claudin-1, leading to hyperuricemia in mice [34,35]. Substantial experimental evidence also supports the observation that LPS serves as an important stimuli of tight junction disruption in a variety of cells, such as intestinal epithelial cells [36] and brain microvascular endothelial cells [37]. Herein, we established tight junction disruption, characterized by a decrease in the expression of tight-junction-related proteins ZO-1, occludin and claudin-1, in IPEC-J2 cells treated with LPS in culture, and the results are also supported by a recent study demonstrating that LPS stimulation led to tight junction disruption in porcine intestines [38].

Rg1 has many protective effects on animal intestinal health, such as alleviating experimental colitis [39], affecting the expression of apoptosis proteins [40] and ameliorating the homeostasis of intestinal flora [41]. In the present study, we confirmed that Rg1 restores the intestinal structure and function by upregulating tight-junction-related proteins ZO-1 occludin and claudin-1 in LPS-induced intestinal dysfunction. These findings are also in line with those of a recent study demonstrating that the integrity of intestinal morphology and tight junction were enhanced in the intestines by the supplementation of Rg1 in the diet of chickens, improving growth performance and intestinal health [42].

Once the tight-junction-protective properties of Rg1 were confirmed, we focused on elucidating the underlying molecular mechanism. According to a recent study, LPS inhibits tight junction expression by activating p38 MAPK in IPEC-J2 cells [38], and we further explored how Rg1 inhibits tight junction disruption focusing on p38 MAPK signaling. Our data showed that Rg1 significantly reduced the phosphorylation of p38 MAPK in LPS-treated IPEC-J2 cells, which might disrupt the downstream signal transduction. It was tested via the pharmacological inhibition of p38 MAPK caused by the specific inhibitor sb203580, investigating whether this interference was associated with the reversal of tight junction disruption in IPEC-J2 cells. SB203580 treatment rescued the expression of the tight-junction-related proteins (ZO-1, occludin and claudin-1) in LPS-treated IPEC-J2 cells. From these results, it has been suggested that the suppression of p-p38 MAPK might be involved in Rg1 inhibition of tight junction disruption. 

*NLRP3*, one of the key regulators of the inflammatory response, mediates a variety of inflammation-associated diseases. It was reported that dextran sulfate sodium (DSS) treatment regulated the expression of *NLRP3* by activating the p38 pathway, resulting in ulcerative colitis [43]; T. gondii-induced *NLRP3* inflammasome activation was strongly associated with the phosphorylation of p38 MAPK in human small intestinal epithelial cells [44]. It has been confirmed that Rg1 ameliorated aging-induced liver fibrosis by inhibiting the *NLRP3* inflammasome in mice [45]. Previous reports showed that SB203580 is a specific inhibitor of p38 MAPK, and widely used in mechanistic studies. It can be inferred that the testing compound plays a biological function by inhibiting p38 MAPK if the compound shows a similar inhibitory effect to SB203580 [46]. Similarly, our data showed that Rg1 inhibited the expression of *NLRP3* and *IL-1β* in IPEC-J2 cells, and the effect was similar to that of SB203580, demonstrating that this inhibition is regulated by p38 MAPK, and this finding is in line with a previous study [47]. 

To further analyze whether *NLRP3* and *IL-1β* are involved in intestinal tight junctions, we employed inhibitors of these two proteins, MCC950 and IL-1Ra, to IPEC-J2 cells, respectively. The results show that these two inhibitors partially blocked the inhibition of LPS on tight-junction-related proteins, namely ZO-1, occludin and claudin-1, indicating that tight junction was regulated by *NLRP3* and *IL-1β*. Our in-vitro data provide evidence that Rg1 can restore the tight junction of *NLRP3* and *IL-1β*, which is regulated by p38 MAPK to some extent. We also investigated whether Rg1 has any effects on tight junction disruption during intestinal dysfunction in mice induced by LPS. This could be helpful for verifying the role of maintaining tight junctions in the interventions for intestinal dysfunction. Our data showed that all the tested tight-junction-related proteins were significantly downregulated in mice with inflamed intestines, suggesting that tight junction disruption occurred. However, Rg1 treatment not only increased the expression of these markers, but also improved the intestinal histology (as demonstrated by hematoxylin–eosin staining). Examinations of p38 MAPK signaling in mice showed that the expressions of p-p38 MAPK, *NLRP3* and *IL-1β* were inhibited by Rg1 treatment, which was consistent with the results obtained in LPS-treated IPEC-J2 cells, confirming the protective molecular mechanism of Rg1.

## 5. Conclusions

In conclusion, the data showed that Rg1 could inhibit intestinal tight junction disruption in LPS-treated IPEC-J2 cells and mice. Rg1 alleviated the increased expression of p38 MAPK-dependent *NLRP3* and *IL-1β* expression and restored the protein expression of occludin, claudin-1 and ZO-1. These results provide evidence supporting the application of an *NLRP3* inhibitor in the treatment of intestinal inflammation. Taken together, these results provided mechanistic insights and scientific evidence to support the use of Rg1 to prevent and treat intestinal tight junction disorders. However, further clinical studies are needed to investigate the protective effects of Rg1 on porcine intestinal inflammation and tight junctions.

## Figures and Tables

**Table 1:** Primers used for qRT-PCR.

Genes	Primer Sequences (5′-3′)	Length (bp)	Accession No.
*NLRP3* (Sus)	F: TCCACACTTCTGACTTCTAAC	241	NM_001256770.2
	R: CCTGCTTCCACCACTACT		
*IL-1β* (Sus)	F: CCCAAAAGTTACCCGAAGAGG	125	NM_214055.1
	R: TCTGCTTGAGAGGTGCTGATG		
*β-actin* (Sus)	F: CTCGATCATGAAGTGCGACGT	114	U07786.1
	R: GTGATCTCCTTCTGCATCCTGTC		
*NLRP3* (Mus)	F: CTGTAACATTCGGAGATTGTGGTT	73	XM_006532858.2
	R: GACCAAGGAGATGTCGAAGCA		
*IL-1β* (Mus)	F: GAAGAAGAGCCCATCCTCTG	98	NM_008361.4
	R: TCATCTCGGAGC CTGTAGTG		
*β-actin* (Mus)	F: CCCTGGAGAAGAGCTACGAG R: TAGTTTCGTGAATGCCGCAG	120	NM_007393.5

The thermocycler was set to 95 °C for 600 s, followed by 40 cycles of 95 °C for 10 s and 60 °C for 30 s. The melting curve was obtained from 65 to 95 °C, increasing in an increment of 0.5 °C every 5 s.

## Data Availability

The data that support the findings of this study are available from the corresponding author upon reasonable request.
